# Traumatic superior semicircular canal dehiscence syndrome: case report and literature review

**DOI:** 10.1093/jscr/rjaa592

**Published:** 2021-01-25

**Authors:** Youssef Aladham, Omar Ahmed, Saad Ahmed Saad Hassan, Elias Francis-Khoury

**Affiliations:** Department of Otolaryngology and Head and Neck Surgery, Poole Hospital NHS Foundation Trust, Poole, UK; Department of Otolaryngology, East Kent Hospitals University NHS Foundation Trust, Ashford, UK; Department of Radiology, Poole Hospital NHS Foundation Trust, Poole, UK; Department of Otolaryngology and Head and Neck Surgery, Poole Hospital NHS Foundation Trust, Poole, UK

**Keywords:** traumatic superior semicircular canal dehiscence, SSCD, head trauma

## Abstract

Superior semicircular canal dehiscence (SSCD) syndrome was first reported in 1998 by Minor *et al.* and comprises a spectrum of auditory and vestibular symptoms as a result of ‘mobile third window’ mechanism. The aetiology of SSCD is debated, but persistent infantile microstructure of the temporal bone was suggested. However, some authors related a ‘second event’, such as closed head trauma, temporal bone fracture and sudden increase in the intracranial pressure to the precipitation of its symptoms. In this article, we report a patient with a closed head trauma who developed unilateral auditory symptoms. High-resolution computed tomography images were obtained and confirmed bilateral SSCD with the normal middle ear structure. The patient was provided with a monaural hearing aid. Literature was searched for similar case reports or series where head trauma precipitated the symptoms of SSCD in anatomically susceptible individuals.

## INTRODUCTION

Superior semicircular canal dehiscence (SSCD) is an inner ear disorder that was first described by Minor *et al.* in 1998 [1]. It is caused by a defect of the bone overlying the superior semicircular canal (SSC) in the floor of the middle cranial fossa resulting in a ‘third window lesion’. The abnormal connection between the middle cranial fossa and inner ear alters the flow of endolymph resulting in a wide variety of auditory and vestibular symptoms, including hearing loss, tinnitus, autophony and vertigo [2]. Vertigo is usually triggered by loud sounds (Tullio phenomenon) or changes in the middle ear pressure (Hennebert’s sign), and hearing loss is commonly low frequency and of conductive type (inner ear conductive hearing loss) [3]. The diagnosis relies on the clinical picture along with enhanced vestibular myogenic potential [4]. However, high-resolution computed tomography (HRCT) of the petrous bone with image reconstruction along the longitudinal axis (**Pöschl view)** and transverse axis (Stenvers view) of the SSC remains a sensitive and popular diagnostic modality [5]. Patients incapacitated by severe vestibular symptoms can have middle fossa or transmastoid plugging or resurfacing of the dehiscent SCC, or less invasively, round window occlusion [6].

## CASE REPORT

A 73-year-old male presented with right-sided reduced hearing and autophony 9 days after sustaining a right-sided closed head trauma as a result of falling downstairs, which also left him with multiple rib fractures and subarachnoid haemorrhage along the inferior surface of the right temporal lobe that was conservatively managed. He had a long history of bilateral tinnitus with no previous hearing difficulty. He denied vertigo and had no previous ear surgery. Initial computed tomography (CT) of the head did not show evidence of skull fracture.

**Figure 1:**
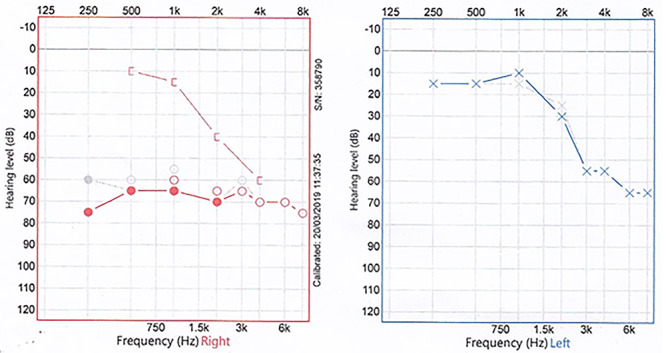
Two superimposed audiograms of the patient, and the interrupted grey line represents the initial one, while the solid coloured line is 5 weeks later; a right mixed hearing loss is noted.

**Figure 2:**
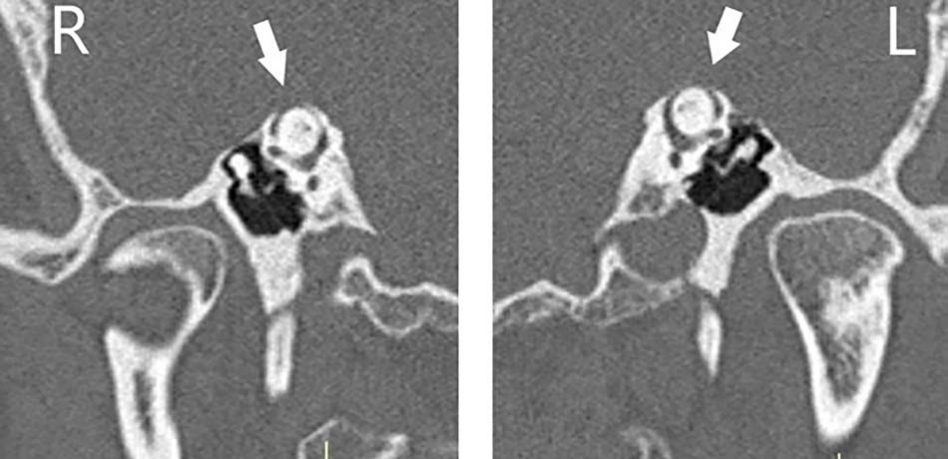
CT scan of the temporal bones of the patient, and images were reformatted in the planes of the right (R) and left (L) SSCs (**Pöschl view)**; bilateral dehiscence (arrows) is noted.

Otoscopic examination was bilaterally normal with no evidence of haemotympanum. At 512 Hz, Weber test lateralized to the right, where Rinne test was found negative. Facial nerve function was normal. An initial audiogram (Fig. 1) demonstrated right severe mixed loss with a maximal air-bone gap of 50 decibel (dB) at 500 Hertz (Hz). There appeared to be a background of bilateral symmetrical high-frequency sloping sensorineural loss, in keeping with presbycusis. Tympanometry was bilaterally type (A). A temporal bone HRCT scan with reformatting in **Pöschl and Stenvers views** was performed and SSCD was found bilaterally (Fig. 2). Ossicular chain, mastoid cells and the rest of the temporal bone were all normal. There was no radiological evidence of ossicular chain disruption. Mastoid cells and the rest of the temporal bone were all normal.

On a follow-up visit 5 weeks later, the patient continued to have the same pattern and degree of hearing loss (Fig. 1). Given the patient’s age and the lack of vestibular symptoms, the patient was provided with a hearing aid.

## DISCUSSION

SSCD is a common radiological finding on temporal bone imaging and is commonly asymptomatic. Carey *et al.* examined 1000 temporal bones microscopically from 596 adults and concluded that true SSCD is found in ~0.4% of adults, of which 20% demonstrated bilaterality. They also reported 1% of adults to have abnormal thinning of the bony coverage of SSC with a higher incidence of bilaterality amongst them [3]. The pathophysiology of SSCD is not yet well understood, with both congenital and acquired processes implicated [3, 7]. It is commonly held that failure of the temporal bone to thicken post-infancy is the main event [3, 8]. As many of those with positive radiological findings remain asymptomatic for several years before they develop clinical symptoms, a role of a ‘second insult’ was suggested [3]. Some authors postulated that event to be sudden change in intracranial pressure such as following closed head injury or even during labour [9]. In 2000, Minor described SSCD in a series of 17 patients, of which 10 patients reported a precipitating event that had led to the onset of their symptoms. Direct head trauma was reported by four patients, while six patients identified the inciting event to be activities that involved changes in intracranial pressure (e.g. straining, violent coughing or lifting heavy objects). These observations suggested that traumatic events may precipitate the symptoms in patients with pre-existing structural abnormalities [2].

We searched the literature for case reports or series of traumatic SSCD syndrome through PubMed interface of Medline database and non-Medline citations searchable with PubMed. As mentioned above, Minor collectively reported four patients with SSCD following direct head trauma [2]. In 2014, Peng *et al.* reported, for the first time, two patients with temporal bone fractures directly causing SSCD where, in both patients, the fracture violated the bony roof of SSC [10]. To our knowledge, there are no other published reports of traumatic SSCD.

We present this case report to reemphasize that patients with acute auditory or vestibular symptoms following head trauma should be systematically evaluated for a third window lesion. Otolaryngologists should be cognizant that closed head trauma with resultant changes in intracranial pressure, as reported here, can acutely precipitate symptoms of SSCD in susceptible individuals, as evidenced by contralateral radiological dehiscence.

## CONFLICT OF INTEREST STATEMENT

The authors state no conflict of interest with regard to the publication of this paper.

## FUNDING

The authors state no funding source for this work.
